# A Macrophage-Derived Factor on Human iPSC-Derived Cardiomyocyte Function: The Role of Osteopontin

**DOI:** 10.3390/cells14231881

**Published:** 2025-11-27

**Authors:** Lei Hao, Eun Jung Lee

**Affiliations:** Department of Biomedical Engineering, New Jersey Institute of Technology, Newark, NJ 07102, USA; lh34@njit.edu

**Keywords:** macrophages, osteopontin, human induced pluripotent stem cell-derived cardiomyocytes (hiPSC-CM), myocardial infarction, inflammation, cardiac remodeling

## Abstract

**Highlights:**

**What are the main findings?**

- Our findings reveal that distinct macrophage subtypes differentially influence the function of hiPSC-CM.
- M2 macrophages secrete significantly high levels of OPN, which can modulate hiPSC-CM function.

**What is the implication of the main finding?**

- Macrophage-derived factors contribute to the communication between macrophages and cardiomyocytes, resulting in functional changes.
- OPN presents a candidate target for therapeutic intervention in cardiac disease.

**Abstract:**

Following MI, massive cardiomyocytes are lost, and inflammatory cells such as monocytes and macrophages migrate into the damaged region to remove dead cells and tissue. While cardiac macrophages are abundant in the injured heart post-MI, the role of inflammation in cardiovascular disease has been under-appreciated in the past. Consequently, the contribution of specific macrophage subsets or macrophage-derived factors on cardiac cells is not well known. Thus, this study investigated the paracrine signaling between human-induced pluripotent stem cell-derived cardiomyocytes (hiPSC-CM) and macrophages, with the focus on the effects of macrophage-derived osteopontin (OPN) on hiPSC-CM function. HiPSC-CM were first co-cultured with unpolarized (M0), pro-inflammatory (M1), or anti-inflammatory (M2) macrophages. The co-culture of hiPSC-CM with M2 macrophages specifically led to notable changes in the electrophysiological properties of hiPSC-CM, including prolonged contraction time (RT90), action potential duration (APD90), and calcium decay time (CSD RT90). Moreover, a significant upregulation of action potential-related genes such as CACNA1C and SCN5A was demonstrated, which coincided with the elevated OPN level in the hiPSC-CM with M2 macrophages co-culture. These functional changes were not observed in the hiPSC-CM-M0 and M1 co-culture groups, likely due to the OPN level remaining below the threshold required to induce detectable changes in hiPSC-CM. Subsequent experiments involving exogenous OPN supplementation and inhibition in hiPSC-CM culture yielded concordant results, further confirming the direct role of OPN in modulating hiPSC-CM gene expression. This study highlights the differential effect of specific macrophage subtypes on hiPSC-CM, as well as the potent bioactivity of OPN and its ability to directly modulate cardiomyocyte behavior, even in the absence of direct cell–cell interactions within a co-culture system. These findings further suggest that OPN could be a novel target for therapeutic intervention in cardiac diseases.

## 1. Introduction

Myocardial infarction (MI) remains one of the leading causes of death worldwide, particularly among aging and metabolically unhealthy populations [1]. MI, commonly known as a heart attack, results from the acute occlusion of coronary arteries [2]. The subsequent oxygen deprivation following MI sets off a complex sequence of events, including cardiomyocyte loss, inflammation, metabolic remodeling, and ultimately fibrotic scarring [3,4]. While some of these responses serve protective or reparative roles, persistent or excessive inflammation and fibrosis progressively compromise cardiac function, often culminating in heart failure—a condition with limited curative options [5]. As such, there is a need to better understand the molecular drivers of post-MI remodeling and to develop novel therapeutic strategies that both preserve cardiomyocyte function and promote tissue regeneration.

One critical but underexploited field in post-MI healing is the interaction of immune cells with cardiac cells and their role in orchestrating inflammation and repair. Macrophages make up most of the immune cell population in the heart, comprising approximately 10% of non-cardiomyocyte cells. During the early inflammatory phase, monocyte-derived macrophages infiltrate the infarct zone and adopt a pro-inflammatory phenotype, producing cytokines such as TNF-α, IL-1β, and IL-6 [6,7]. These cytokines amplify inflammation and facilitate the clearance of necrotic tissue [8]. As healing progresses, macrophages transition to reparative phenotypes that secrete anti-inflammatory mediators like IL-10 and TGF-β. The anti-inflammatory macrophages support angiogenesis and promote ECM remodeling and fibroblast activation [6,7].

Recent studies have revealed the diverse population of macrophages and their roles in cardiovascular tissues. One study reported that bone marrow-derived CD72-positive macrophages exacerbate cardiac injury in a mouse model, underscoring their contribution to disease progression and suggesting the therapeutic potential of targeting specific macrophage subpopulations within the cardiac microenvironment [9]. Another study showed that CCR2^+^ monocyte-derived macrophages dominate early post-MI inflammation, whereas CCR2^−^ tissue-resident macrophages play cardioprotective roles by promoting angiogenesis [10]. It has also been shown that the macrophages regulate endothelial cell proliferation [11], influence fibroblast differentiation [12], and contribute to vascular network formation [13]. These intercellular interactions help orchestrate the complex healing response post-MI. While more is known about macrophage crosstalk with fibroblast and vascular cells, their direct interactions with surviving cardiomyocytes remain less well defined. Moreover, the contribution of specific macrophage subsets or macrophage-secreted factors on cardiac cell function is not fully understood.

To address the gap in knowledge, our previous study examined the effects of different macrophage subsets on the function of cardiomyocytes derived from mouse embryonic stem cells (mESC-CM) [14]. Specifically, the impact of macrophages on cardiomyocyte calcium handling was demonstrated using a co-culture system. It was demonstrated that both pro- and anti-inflammatory macrophages secrete osteopontin (OPN) in monoculture and in co-culture with mESC-CM. Additionally, the inhibition of macrophage-secreted OPN led to a noticeable reduction in the expression of store-operated calcium entry (SOCE), suggesting that macrophage-derived OPN could play an important role in cardiac function, especially in its Ca^2+^ handling function. OPN is a matricellular protein first identified in bone tissue but shown to be expressed by various other cell types such as smooth muscle cells [15], epithelial cells [16], and macrophages [17,18]. In the cardiac tissue, the expression of OPN is normally almost absent, but peaks during the early inflammatory phase within 7 days post-MI [19,20]. Moreover, the plasma level of OPN is shown to be associated with the severity of heart failure in dilated cardiomyopathy patients [21] and serves as a potential biomarker for predicting cardiovascular events, particularly among individuals diagnosed with type 1 diabetes [22]. Prolonged elevation and overexpression of OPN may have detrimental effects, as excessive myocardial OPN expression is also recognized as an indicator of adverse cardiac remodeling and fibrosis [23]. While these studies provide evidence that OPN plays a vital role in cardiac function or protection, the precise role of OPN in the post-MI microenvironment, particularly its interactions with cardiac cells and its specific connection to cardiac disease following MI, remains to be elucidated. Therefore, building on our previous findings, this study further investigated the effects of macrophages and macrophage-secreted OPN on human-induced pluripotent stem cell-derived cardiomyocytes (hiPSC-CM) function.

## 2. Materials and Methods

### 2.1. Human-Induced Pluripotent Stem Cell (hiPSC) Culture

HiPSCs (SFLB6 line, LUMC0020ICTRL-06) were cultured and maintained as previously described [24]. Briefly, hiPSCs were seeded on Vitronectin coated six-well plates (125,000 cells/well) in Essential 8 (E8) media (Thermo Fisher, Waltham, MA, USA). Cells were sub-cultured once they reached approximately 70% confluency, with passages occurring every three to four days.

### 2.2. Differentiation of HiPSC into Cardiomyocytes

HiPSCs were differentiated into hiPSC-cardiomyocytes (CM) following an established protocol [25]. Briefly, hiPSCs (250,000 cells) were seeded onto Matrigel coated six-well plates on day −1. For cardiac mesoderm induction on day 0, the culture medium was changed from E8 to BPEL (Bovine Serum Albumin Polyvinyl Alcohol Essential Lipids) containing a combination of bone morphogenetic protein 4 (BMP4, 20 ng/mL), ACTIVIN A (20 ng/mL), and a small-molecule inhibitor of glycogen synthase kinase-3β (CHIR 99032, 1.5 μM). Subsequently, WNT signaling was inhibited using XAV939 (5 μM) on days 3 to 6 of differentiation. Culture medium was then replaced with fresh BPEL on day 6 and subsequently changed every 3 or 4 days until differentiation was completed.

### 2.3. HiPSC-CM Dissociation and Enrichment

On day 20–21 of differentiation, hiPSC-CM were dissociated from the plates and collected as suspension using an enzymeT dissociation kit (Miltenyi Biotec, Bergisch Gladbach, Germany). To further enrich the hiPSC-CM population and remove non-CM population, cells were subjected to magnetic sorting following the manufacturer’s instructions (Miltenyi Biotec, Bergisch Gladbach, Germany). Briefly, the cell pellet was resuspended in FACS buffer (PBS with 2% fetal bovine serum) and incubated with a non-CM Depletion Cocktail (20 µL, Miltenyi Biotec, Bergisch Gladbach, Germany) at 4 °C for 5 min. After centrifugation, the pellet was resuspended in FACS buffer and incubated with the Anti-Biotin microbeads (20 µL, Miltenyi Biotec, Bergisch Gladbach, Germany) at 4 °C for 10 min. HiPSC-CM was collected through magnetic separation and further enriched using a CM Enrichment Cocktail (Miltenyi Biotec, Bergisch Gladbach, Germany).

### 2.4. Characterization of HiPSC-CM

To assess the purity of dissociated hiPSC-CM, FACS (MacsQuant Analyzer 10, Miltenyi Biotec, Bergisch Gladbach, Germany) analysis was performed. Cells were stained with 5 μL of VioBlue Anti-Cardiac Troponin T antibody (BD Biosciences, San Jose, CA, USA) for 45 min at 4 °C and resuspended in the autoMACS Running Buffer (Miltenyi Biotec, Bergisch Gladbach, Germany) after multiple washes in the permeabilization buffer. Unstained cells and an isotype control were used as negative controls. To visualize the sarcomeric organization, fixed hiPSC-CM were incubated with a monoclonal mouse anti-α-Actinin antibody (1:800, A7811, MilliporeSigma, Rockville, MD, USA) followed by a donkey anti-mouse secondary antibody (1:100, 715-165-15, Biozol, Hamburg, Germany). Samples were examined and imaged using a confocal laser fluorescent microscope (Leica SP8 WLL, Wetzlar, Germany).

### 2.5. HiPSC-Macrophage Culture and Differentiation

HiPSCs were successfully differentiated into macrophage subsets following a previously established protocol by Cao et al. [26]. Briefly, undifferentiated hiPSCs were cultured on Matrigel-coated plates with mTeSR-E8 medium on day −1. On day 0, IF9S media supplemented with BMP4 (25 ng/mL), ACTIVIN A (15 ng/mL), and CHIR 99021 (1.5 µM) were used. On day 2, media containing cytokines such as VEGF (50 ng/mL), FGF2 (50 ng/mL), SCF (50 ng/mL), and SB431542 (10 µM) were used. On day 5, cytokines supplemented in the medium were switched to VEGF (50 ng/mL), FGF2 (50 ng/mL), SCF (50 ng/mL), TPO (50 ng/mL), IL3 (10 ng/mL), and IL6 (50 ng/mL). The cytokines were then switched to IL3 (10 ng/mL), IL6 (50 ng/mL), and M-CSF (80 ng/mL) on day 9. CD4 positive monocytes were isolated on day 14 or day 15 via magnetic isolation. The isolated cells were cultured in IF9S media supplemented with M-CSF (80 ng/mL) for 4 days, resulting in the differentiation into M0 macrophages. To induce further differentiation into M1 or M2 macrophages, IFNγ and LPS (10 ng/mL) or IL-4 (20 ng/mL) were added, respectively, for an additional two days.

### 2.6. HiPSC-CM and HiPSC-Macrophages Co-Culture

Dual-well open-bottom silicone inserts were placed on the fibronectin-coated (10 μg/mL, Sigma-Aldrich, Burlington, MA, USA) tissue culture plates (Ibidi, Fitchburg, WI, USA). In one of the wells of the insert, hiPSC-macrophages (70 K cells/well) were seeded and polarized into subtypes. In the adjacent well, hiPSC-CM (200 K cells/well) were seeded. A co-culture of hiPSC-CM and hiPSC-macrophages was initiated by removing the inserts, which created a cell-free gap of 500 µm between the two cell types.

### 2.7. RT-PCR

Total RNA was extracted and purified from hiPSC-CM and hiPSC-macrophages of all experimental groups using a GenElute Mammalian Total RNA Miniprep Kit (Sigma-Aldrich, Burlington, MA, USA) following the manufacturer’s instructions. The cDNA was synthesized using an iScript cDNA Synthesis Kit (Bio-rad, Hercules, CA, USA). The RT-PCR was performed using a T100 Thermal Cycler (Bio-rad, Hercules, CA, USA), as described previously [14]. To examine the phenotype of hiPSC-macrophages, the expression of *IL6*, *IL8*, and *CXCL10* for M1, *CD206*, *CD163*, and *CD200R* for M2, and *CD68* and *CD29* for M0 (non-specific) were evaluated. For hiPSC-CM, the expression of genes involved in sarcomere assembly (*MYL2*, *MYL7*, *MYL4*, *MYL3*, *MYH6*, *MYH7*, *ACTN2*), in cardiac action potential (*SCN5A*, *CACNA1C*, *KCNQ1*, *KCNE1*, *KCNH2*, *KCNJ12*, *KCNJ2*, *HCN4*), and in calcium-handling (*NCX1*, *SERCA2A*, *CASQ2*) was examined. *RPL37A* and *HARP* were used as housekeeping genes. The results were analyzed by a Comparative CT (ΔΔCT) method.

### 2.8. Triple Transient Measurement (TTM)

The TTM system was used to determine the functional properties of cells, as previously described [27]. The TTM system captures the signals of action potential, calcium, and contraction simultaneously, allowing a comprehensive analysis of cardiac function. HiPSC-CM were incubated with ANNINE 6-plus (stock concentration: 0.7 mM, dilution 1:833, Sensitive Farbstoffe GbR, Munich, Germany), Rhod 3 (stock concentration 10 mM, dilution 1:833, Invitrogen, Waltham, MA, USA) and CellMask Deep Red (stock concentration 5 mg/mL, dilution 1:1000, Thermo Fisher Scientific, Waltham, MA, USA) in BPEL medium for 20 min at 37 °C, refreshed, and given 10 min to recover at 37 °C before recording baseline measurements. Then, an all-optical fluorescent system recorded sequential frames while switching wavelengths for 1 millisecond exposure at 470 nm, 560 nm, and 656 nm. A multi-bandpass filter was used to capture the respective emissions each millisecond. Data were analyzed automatically using custom software offline to reduce user bias.

### 2.9. OPN Experiments

OPN secretion by hiPSC-macrophages, hiPSC-CM, and in a co-culture after 24 h was quantified using a Human Osteopontin Quantikine ELISA (R&D Systems, Minneapolis, MN, USA), following manufacturer’s instructions. For OPN addition studies, recombinant OPN (500 ng/mL, R&D Systems, Minneapolis, MN, USA) was supplemented to hiPSC-CM culture for either 24 or 72 h. HiPSC-CM cultured without OPN supplement served as a control. The 24 h duration aligns with the co-culture experiments, which enables the examination of the immediate effects of OPN on hiPSC-CM function. The 72 h duration was performed to observe the sustained impact of OPN. Moreover, an OPN-specific neutralizing antibody (1 μg/mL, R&D Systems, Minneapolis, MN, USA) was added to the culture for either 24 or 72 h to inhibit OPN. The expression of CD44 by hiPSC-CM (anti-human CD44 antibody, 1:20 dilution, Miltenyi Biotec, Charlestown, MA, USA) was evaluated by FACS analysis. Unstained cells and isotype control served as negative controls.

### 2.10. Cell Migration

Time-lapse experiments were performed using an EVOS Cell Imaging system (EVOS FL AUTO2, Thermo Fisher Scientific, Waltham, MA, USA). The imaging took place in a humidified chamber with a controlled environment at 37 °C and 5% CO_2_. Differential interference contrast images were captured at 20 min intervals continuously over 12 h. ImageJ 1.54j was used to analyze cell migration from the acquired images.

### 2.11. Statistical Analysis

Results are presented as mean ± standard deviation. Statistical analysis was performed using a two-tailed independent samples *t*-test, a one-way ANOVA, or their nonparametric counterpart, followed by Tukey and Games–Howell post hoc tests, where appropriate. Statistical significance was accepted for * *p* < 0.05.

## 3. Results

**Figure 1:**
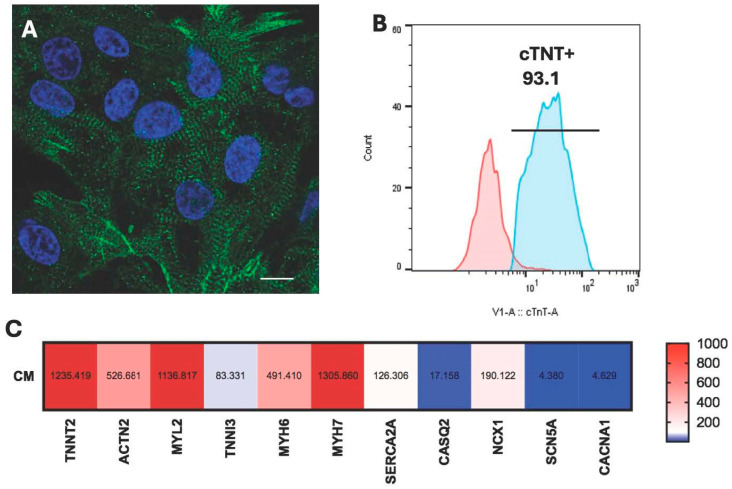
Characterization of hiPSC-CM. (**A**) A fluorescence image of dissociated hiPSC-CM stained with α-Actinin, revealing well-developed sarcomeres in green and nuclei in blue. Scale bar = 10 μm. (**B**) Flow cytometry analysis demonstrating highly purified (>93%) cTnT positive hiPSC-CM. (**C**) RT-PCR results displaying the expression profile of cardiac-specific genes in hiPSC-CM.

**Figure 2:**
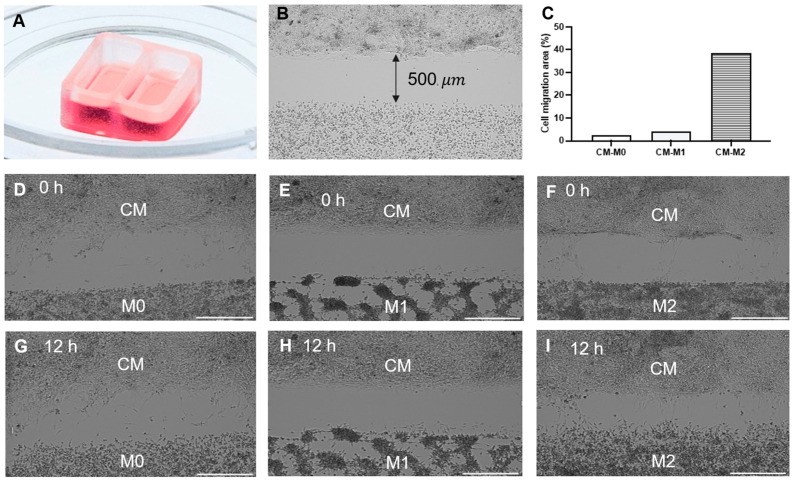
Co-culture of hiPSC-CM with hiPSC-derived macrophages. (**A**) Two-well silicone insert used for initial seeding of hiPSC-CM and hiPSC-derived macrophages. (**B**) A 500 μm wide gap between cell types was created after the silicon insert was removed. (**C**) Quantitative analysis of cell migration after 12 h of co-culture. (**D**–**F**) Time-lapse phase-contrast images of hiPSC-CM with hiPSC-M0, M1, and M2 at time 0 and 12 h (**G**–**I**). Scale bar = 500 µm.

Both cardiomyocytes and macrophages were successfully differentiated from hiPSCs using a well-established protocol [25,26]. The spontaneously beating cells were detected after 10–12 days since the start of the differentiation and highly purified hiPSC-CM were successfully obtained and characterized after 20–21 days of differentiation. The immunofluorescence images revealed the presence of well-developed sarcomeres in hiPSC-CM, characterized by the striated and organized structure (Figure 1A). The flow cytometry analysis confirmed the efficiency of the differentiation, with highly enriched cTnT-positive cardiomyocytes (>93%) in the collected cell population (Figure 1B). RT-PCR analysis further demonstrated the robust expression of key cardiac-specific genes such as *TNNT2, ACTN2*, *MYL2*, *MYH6*, and *MYH7* (Figure 1C). To investigate the interactions, hiPSC-CM and macrophage subsets (M0, M1, and M2), were seeded in the silicone inserts (Figure 2A), which created a 500 μm cell-free gap upon seeding. Once the cells were attached and formed a monolayer, the inserts were removed, allowing the two cell types to share the medium (Figure 2B). Time-lapse images acquired over a 12 h period were analyzed using ImageJ software to quantify cell migration. The changes in the area covered by cells over time were measured and quantified. The results showed that hiPSC-CM, as well as hiPSC-M0 and hiPSC-M1 macrophages, remained relatively stationary and maintained their respective morphology even after 12 h in the co-culture. The coverage area of hiPSC-M0 and hiPSC-M1 macrophages was increased only by 2.4% and 4.2%, respectively. HiPSC-M2 macrophages, on the other hand, exhibited the greatest translocation towards hiPSC-M2, displaying approximately 38.4% more coverage compared to its initial state (Figure 2C–I).

**Figure 3:**
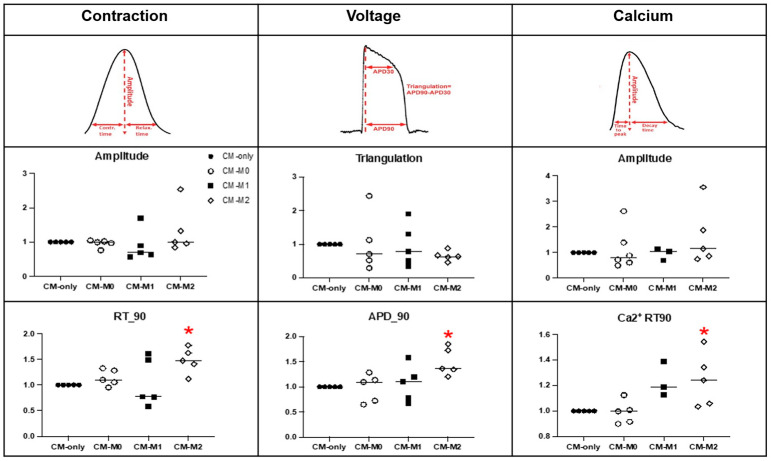
Functional assessment of hiPSC-CM from the co-culture by the TTM system. The TTM data demonstrate the alteration of electro-physiological properties in hiPSC-CM co-cultured with macrophage subtypes (*n* = 5, one-way ANOVA, * *p* < 0.05).

**Figure 4:**
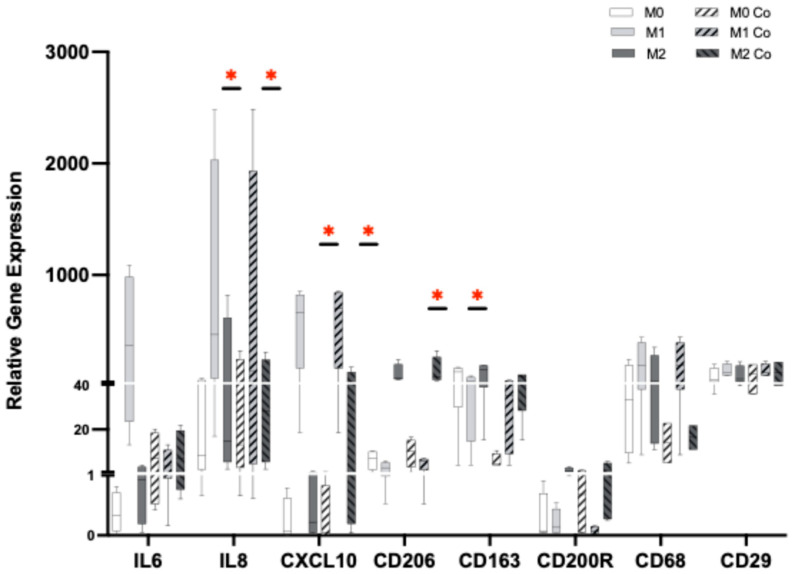
Gene expression analysis of hiPSC-macrophages. RT-PCR analysis revealed significantly high expression of macrophage-subtype-specific transcriptional genes such as *IL-8* and *CSCL10* in hiPSC-M1 macrophages and *CD206* in hiPSC-M2 macrophages, validating their corresponding phenotypes. *CD68* and *CD29* were used as nonspecific markers to macrophage polarization. No significant changes were observed in the expression even after 24 h of co-culture with hiPSC-CM (*n* = 4, one-way ANOVA, * *p* < 0.05).

In addition to the migration assay, the function of hiPSC-CM co-cultured with macrophage subtypes was assessed using a TTM system which provides simultaneous recordings of contraction, action potential, and calcium flux signals. Independent of macrophage subtypes, hiPSC-CM exhibited a similar amplitude of contraction in all groups (Figure 3). However, hiPSC-CM co-cultured with M2 macrophages displayed a significantly prolonged contraction–relaxation time to 90% (RT90), indicating a delayed mechanical relaxation phase in the cardiomyocytes. In addition, while no differences were observed in the triangulation of action potential duration (APD90-APD50), hiPSC-CM co-cultured with hiPSC-M2 macrophages exhibited a significantly extended action potential duration at 90% (APD90). HiPSC-CM also showed a significant increase in the Ca^2+^ decay time (CSD RT90) when co-cultured with hiPSC-M2 macrophages, while the amplitude of the Ca^2+^ decay time signal remained similar among the groups. To confirm the phenotype of macrophage subsets in the co-culture, gene expression analysis was performed (Figure 4). As expected, hiPSC-M1 macrophages significantly expressed IL-8 and CSCL10, while hiPSC-M2 macrophages significantly expressed CD206. All M0, M1, and M2 macrophages expressed CD68 and CD29, which were used as nonspecific markers for macrophage polarization. No significant changes were observed in their expression, validating their phenotype during the 24 h of co-culture with hiPSC-CM (*n*= 4, one-way ANOVA, * *p* < 0.05).

**Figure 5:**
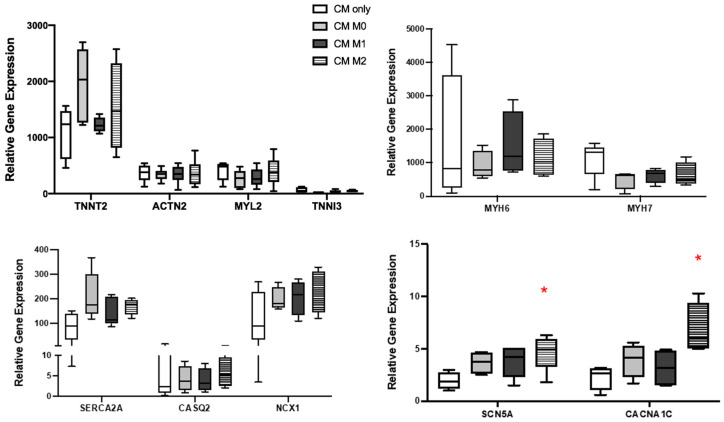
Gene expression analysis of hiPSC-CM in co-cultures. RT-PCR analysis revealed a significant upregulation of the cardiac ion channel genes such as *SCN5A* and *CACNA1C* in the hiPSC-CM-M2 macrophage co-culture group. Structural and contractile genes such as *TNNT2, ACTN2, TNNI3,* and *MYL2* showed no significant differences among the groups. Similarly, calcium handling genes such as *SERCA2A, CASQ2,* and *NCX1* exhibited comparable expression levels across conditions. No significant changes were observed in the expression of myosin isoform genes such as MYH6 and MYH7 (*n* = 5, one-way ANOVA, * *p* < 0.05).

To better understand whether these functional alterations in hiPSC-CM are associated with gene expression, RT-PCR was performed on hiPSC-CM after 24 h of co-culture with macrophages as well. The results showed that the expression of TNNT2, ACTN2, TNNI3, MYL2, NCX1, MYH6, and MYH7 remained similar across all experimental groups (Figure 5). However, the co-culture with hiPSC-M2 macrophages resulted in a significant upregulation of genes such as calcium voltage-gated channel subunit alpha1C (CACNA1C) and sodium voltage-gated channel alpha subunit (SCN5A) in hiPSC-CM.

**Figure 6:**
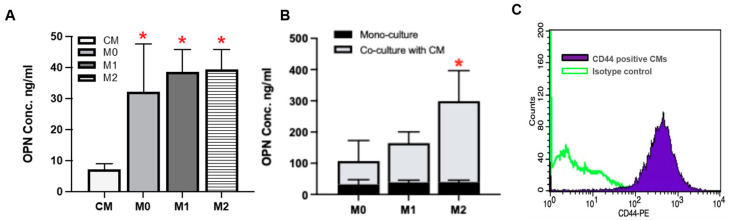
OPN secretion profile by hiPSC-CM and hiPSC-derived macrophage subtypes. (**A**) OPN secretion was significantly higher in monoculture of hiPSC-derived macrophages compared to hiPSC-CM after 24 h in culture (*n* = 5, one-way ANOVA, * *p* < 0.05). (**B**) When in co-culture, a significantly higher OPN secretion was observed only in the hiPSC-CM-M2 co-culture group compared to all the other groups (*n* = 3, one-way ANOVA, * *p* < 0.05). (**C**) CD44 expression by hiPSC-CM was confirmed by a flow cytometry analysis.

Since the cells did not come into direct contact with each other in the co-culture, as demonstrated in the migration assay, it was speculated that the observed functional changes in hiPSC-CM are mediated by paracrine signaling. Given that our previous study suggested a potential role of macrophage-secreted OPN, the level of OPN secretion by cells was first examined. As expected, hiPSC-CM exhibited a negligible amount of OPN. OPN secretion by monocultured macrophages, however, was significantly higher than that of hiPSC-CM, with levels ranging from 20 to 50 ng/mL (Figure 6A, * *p*< 0.05). Interestingly, the co-culture of hiPSC-CM and macrophages resulted in a marked upregulation of OPN secretion in all of the experimental groups. Notably, the hiPSC-CM and hiPSC-M2 macrophage co-culture exhibited significantly higher OPN secretion compared to other co-culture groups, as well as monocultured hiPSC-CM and hiPSC-macrophage subtypes (Figure 6B, * *p*< 0.05)

**Figure 7:**
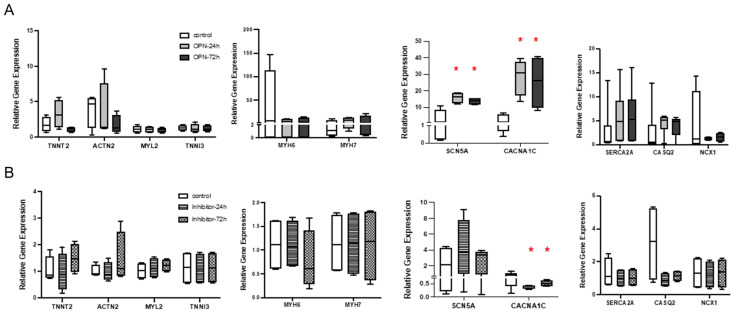
The effects of exogenous OPN and OPN inhibitor on hiPSC-CM gene expression. (**A**) RT-PCR results show that treatment with recombinant OPN for 24 and 72 h led to a significant upregulation of *SCN5A* and *CACNA1C* expression in hiPSC-CM, mirroring the pattern observed in the co-culture experiments. (**B**) Treatment with an OPN inhibitor for 24 and 72 h resulted in a significant downregulation of *CACNA1C* (*n* = 4, one-way ANOVA, * *p* < 0.05).

To further investigate the direct role of OPN, exogenous OPN was added into the hiPSC-CM culture instead of the co-culture with macrophages. The potential binding and interaction between hiPSC-CM with OPN was confirmed by the expression of CD44, which is identified as one of the OPN receptors (Figure 6C). OPN treatment for 24 or 72 hrs did not elicit changes in the expression level of *TNNT2, ACTN2, TNNI3, MYL2, NCX1, MYH6,* or *MYH7* genes in all experimental groups. However, consistent with the findings from the hiPSC-CM and hiPSC-macrophage co-culture experiments, a significant upregulation of *CACNA1C* and *SCN5A* was observed at both time points (Figure 7A, * *p* < 0.05). Further, the upregulation of *CACNA1C* and *SCN5A* was reversed with the addition of an OPN inhibitor, indicating OPN as an effector. In fact, a significant downregulation of *CACNA1C* compared to the control group observed with the OPN inhibitor further supports a potential direct regulatory relationship between OPN and *CACNA1C* (Figure 7B, * *p* < 0.05).

## 4. Discussion

Within the heart, macrophages have increasingly been recognized as one of the key cell types involved in various physiological and pathological processes that influence cardiac function [28]. A large number of macrophages are present in the normal heart and an average of 4–5 macrophages are in direct contact with each cardiomyocyte [8]. The interaction between macrophages and cardiac cells is complex as they can communicate through direct cell–cell interactions as well as via secreted proteins through paracrine signaling [29,30]. Cell–cell interactions result in the changes in macrophage phenotype [31,32], cardiomyocyte morphology, or electrical signaling [33] with either positive or negative effects on cardiac homeostasis and diseases [34]. A previous study by Hulsmans et al. demonstrated that cardiac resident macrophages are involved in facilitating cardiac electrical conduction via the distal atrioventricular node [35]. It is also known that both pro-inflammatory and anti-inflammatory macrophage subsets release a diverse range of cytokines [36,37]. These macrophage-secreted factors can influence the development of conditions conducive to arrhythmias and affect cardiac electrophysiology [38,39,40]. Monnerat et al. indicated a direct link between macrophage-mediated inflammatory response and changes in the electrophysiological properties of myocardial tissue. It was shown that increased IL-1β secretion by macrophages led to a prolongation of the APD in mouse ventricular myocardial strips in their ex vivo study [41]. Another study also demonstrated that the polarization of macrophages prolongs APD in cardiomyocytes via gap junction and KCa3.1 activation, which can lead to post-MI arrhythmias [42]. However, the exact role of different macrophage subsets and macrophage-secreted factors on cardiomyocytes has not been fully determined.

Thus, extended from our previous study which demonstrated that macrophages influence the Ca^2+^ handling mechanism of mESC-CM and identified a potential role of OPN in modulating cardiac cell function [14], the present study investigates how macrophages affect the function of hiPSC-CM through paracrine signaling. Consistent with our previous findings from RAW264.7 cells and mESC-CM [14], all subtypes of hiPSC-derived macrophages secreted significantly higher levels of OPN than hiPSC-CM. The OPN secretion was further elevated in the hiPSC-CM-M2 macrophage co-culture, which was associated with notable changes in electrophysiological properties and a significant upregulation of *SCN5A* and *CACNA1C* gene expression. SCN5A regulates the influx of Na^+^ [43] while *CACNA1C* encodes a voltage-gated Ca^2+^ channel that mediates Ca^2+^ entry during the cardiac action potential and is implicated in arrhythmia when mutated [44,45]. Thus, the upregulation in these gene expressions likely enhances both Ca^2+^ and Na^+^ influx, potentially contributing to the observed alterations in the electrophysiological properties of cardiomyocytes.

A notable increase in the OPN secretion in the co-culture of hiPSC-CM and M2 macrophages, together with concurrent changes in hiPSC-CM function, indicates OPN as one of the critical contributing factors. Since the two cell types did not come into direct contact in our co-culture model, it is likely that the observed changes in cell function resulted from paracrine signaling. This finding is further supported by the addition of recombinant OPN into hiPSC-CM culture, which led to a significant upregulation of CACNA1C and SCN5A genes, mirroring the patterns observed in the co-culture of hiPSC-CM and M2 macrophage. The altered expression of CACNA1C observed in OPN-supplemented groups aligns with the finding from a previous study demonstrating that OPN is intricately linked to Ca^2+^ influx mechanisms [46]. Subsequent OPN inhibition experiments yielded consistent results, further confirming the direct role of OPN in modulating hiPSC-CM gene expression. As these functional changes were not observed in the hiPSC-CM-M0 and M1 co-culture groups, the differential effects of macrophage subsets on cardiomyocyte function are substantiated. Moreover, the observed increase in OPN secretion by M2 macrophages in response to cardiomyocyte suggests a dynamic and potentially adaptive regulatory mechanism, wherein macrophages adjust their secretory profile based on the microenvironmental cues [47].

**Figure 8:**
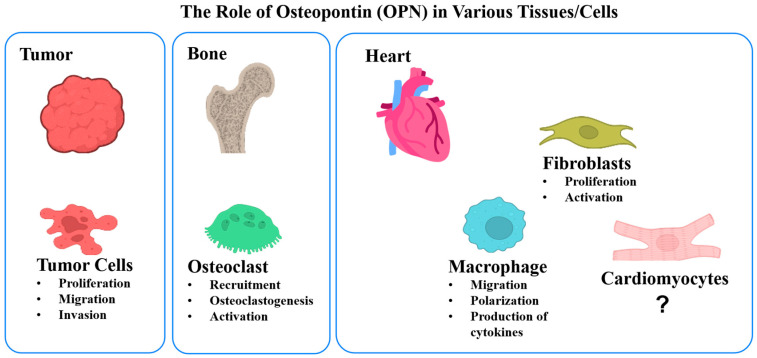
The effects of OPN on various tissues and cell types. In tumor cells, OPN promotes proliferation, migration, and invasion, contributing to tumor progression. Within bone tissue, OPN regulates osteoclast recruitment, differentiation (osteoclastogenesis), and activation, influencing bone homeostasis. In cardiac tissue, OPN plays a role in cardiac fibroblast proliferation and activation, leading to cardiac fibrosis and post-MI healing. OPN also modulates macrophage behavior, influencing their migration, polarization, and cytokine production that are critical for cardiac inflammatory responses and tissue healing. The specific effect of OPN on cardiomyocytes is not well known and awaits further investigation.

OPN is a multifunctional protein present in various tissues [17] and has been implicated in regulating the function of different cell types across tissues, as illustrated in Figure 8 [4,48,49,50]. In the bone, OPN is associated with tumor development, progression, and metastasis [51,52], as well as with bone remodeling [53]. In the heart, OPN is critical for myofibroblast differentiation, which is required for scar formation during cardiac remodeling [54]. Typically, under physiological conditions, there is a circulating level of OPN in the vasculature range between approximately 10 ng/mL and 37 ng/mL [55,56]. Upon cardiovascular injury, however, profound upregulation of OPN is detected [57]. OPN primarily originating from infiltrating macrophages is localized in the infarcted areas and contributes to macrophage polarization, migration, and cytokines production [17,58]. Our findings indicate that the administered OPN concentration (500 ng/mL) was adequate to independently replicate the biological effects observed in the hiPSC-CM-M2 co-culture condition. The selected OPN dose used in our experiments falls within the range of concentrations (180–5000 ng/mL) reported in previous studies, which measured OPN levels either from cultured cells, or in plasma or tissue following cardiovascular damage [59,60]. Since the OPN levels measured in the hiPSC-CM-M0 and hiPSC-CM-M1 groups were considerably lower than that of the hiPSC-CM-M2 macrophages, it is speculated that the OPN levels in the hiPSC-CM-M0 and M1 groups were insufficient and below the threshold required to induce detectable functional changes in hiPSC-CM.

Furthermore, the presence of one of the OPN receptors, CD44, in hiPSC-CM validated the capacity of these cells to respond to OPN. CD44 is considered as a preferred OPN receptor in immune cell studies [61,62], as the binding of OPN to CD44 is crucial for modulating immune responses, including macrophage-driven processes [63]. The interaction of OPN with CD44 is also shown to mediate critical processes in the cardiac inflammation, fibrosis, and cardiac remodeling [64].

In summary, this study highlights the differential effect of specific macrophage subtypes on hiPSC-CM as well as the potent bioactivity of OPN and its ability to directly modulate cardiomyocyte behavior, even in the absence of direct cell–cell interactions within a co-culture system. While the current study focused on the short-term effects of OPN on healthy cardiomyocyte function, it would be valuable to investigate the long-term effects of elevated levels of OPN as well as the effect of OPN on injured cardiomyocyte and repair. Previous clinical studies have shown the positive effects of an acute increase in OPN level on wound healing and angiogenesis [65,66]. On the other hand, a chronic increase in OPN has been identified as a strong predictor of an unfavorable prognosis for major adverse cardiovascular events, regardless of the presence of traditional risk factors [23,64]. Future studies are needed to clarify the mechanisms by which OPN transitions from a protective to a pathological role over time, particularly in relation to fibrosis, inflammation, and cardiomyocyte remodeling. Furthermore, in vivo longitudinal studies which allow the tracking of disease progression and treatment effects over time will be valuable for evaluating the therapeutic potential of modulating OPN levels at different stages of cardiovascular disease.

## 5. Conclusions

This study demonstrates the effects of macrophage-secreted OPN on hiPSC-CM function. The co-culture of M2 macrophages and hiPSC-CM led to a significantly elevated secretion of OPN, which resulted in the alterations of electrophysiological properties and key ion channel gene expressions of hiPSC-CM. Supplementation and inhibition experiments further confirmed a direct regulatory role of OPN on cardiomyocytes, underscoring its importance as a paracrine factor in macrophage–cardiomyocyte interactions and its potential as a therapeutic target for post-injury cardiac remodeling.

## Data Availability

The raw data supporting the conclusions of this article will be made available by the authors on request.
